# Asymptomatic Bradycardia and Normal Chest Radiography in a Patient With Spontaneous Pneumomediastinum: A Case Report of a Rare Entity

**DOI:** 10.7759/cureus.73883

**Published:** 2024-11-17

**Authors:** Md Shamsuzzaman, Deepika Chhabra, Koshy Thomas

**Affiliations:** 1 Acute Internal Medicine and General Internal Medicine, East Suffolk and North Essex NHS Foundation Trust, Ipswich, GBR; 2 Acute Internal Medicine, Mid and South Essex NHS Foundation Trust, Essex, GBR

**Keywords:** a rare case, bradycardia, chest ct scan, chest radiography, spontaneous pneumomediastinum (spm)

## Abstract

Spontaneous pneumomediastinum (SPM) is an uncommon condition caused by alveolar rupture due to increased intra-alveolar pressure resulting in air tracking along the tracheobronchial tree. While chest pain, neck pain, and dyspnea are the most commonly described symptoms, bradycardia could be an associated manifestation occasionally. In the majority of cases, pneumomediastinum is usually diagnosed on chest X-ray. We present an atypical case of chest X-ray-negative SPM complicated by marked bradycardia, which has been sparsely documented in published literature.

## Introduction

Pneumomediastinum, less frequently referred to as mediastinal emphysema, develops when air extravasates from within the airways, lungs, or esophagus and migrates into the mediastinal space. Spontaneous pneumomediastinum (SPM) is typically a benign, self-limiting condition that occurs in otherwise healthy subjects without any obvious identifiable cause; however, there are some reported predisposing factors, which may include smoking, recreational drug inhalation, pre-existing obstructive, or restrictive airway disease. Additionally, strenuous physical activity, bouts of cough, retching, and vomiting have also been reported as triggering events [1]. The other category of pneumomediastinum, with clear traumatic or iatrogenic causative factors, is referred to as secondary pneumomediastinum. A plain chest X-ray usually depicts positive findings consistent with a diagnosis; however, if findings are equivocal, a CT scan of the chest can definitively rule in or rule out the diagnosis. We report a case that turned out to be complex in terms of diagnosis due to non-diagnostic initial chest radiography coupled with an unusual complication of marked bradycardia, which is a rare entity.

## Case presentation

**Figure 1:**
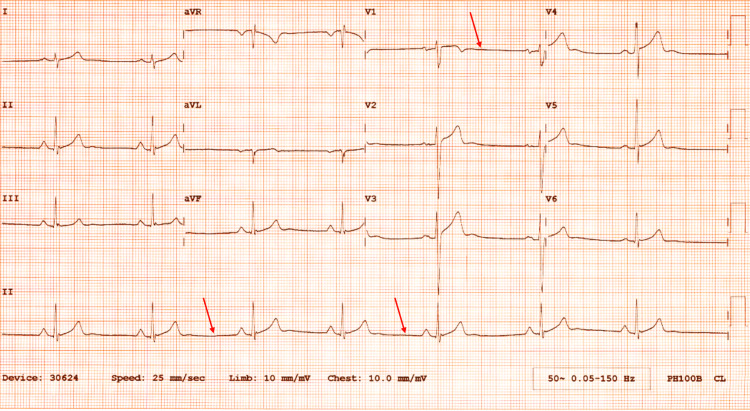
ECG depicting sinus bradycardia ECG: electrocardiogram

A young male in his early-20s presented with insidious-onset shortness of breath and pleuritic chest pain which was radiating to his neck and shoulder. He denied any fever, cough, dysphagia, or vomiting. There was no history of respiratory illness, cardiac disease, or connective tissue disorder. The patient did not smoke or consume alcohol. He had never used illicit drugs. On admission, the physical examination was unremarkable except for significant bradycardia with a pulse rate of 38 per minute. His oxygen saturation was 98% on room air. ECG showed sinus bradycardia without ST-T changes (Figure 1), and no acute abnormalities were noted on plain chest X-ray. Routine blood tests including thyroid functions were reported to be normal.

**Figure 2:**
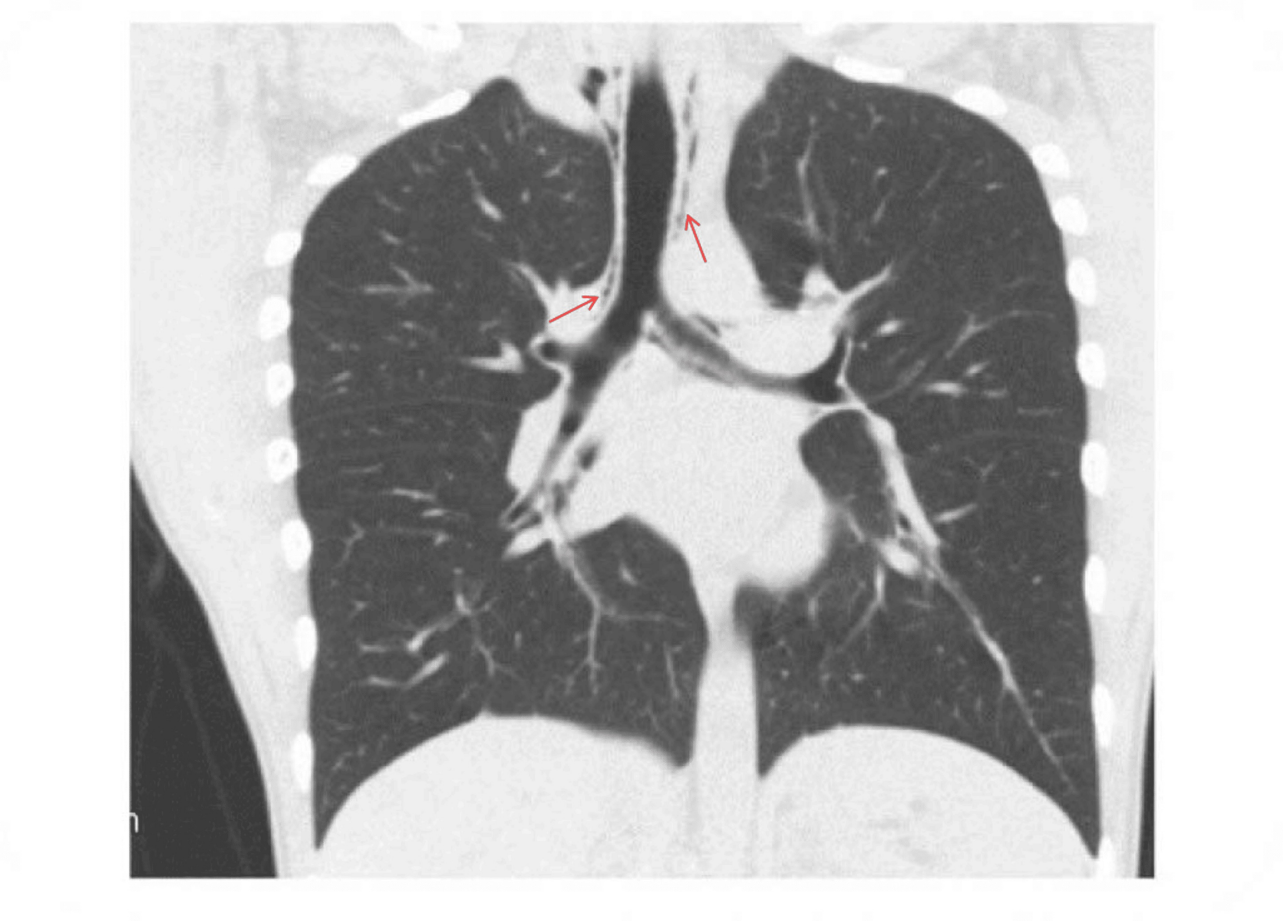
CT chest demonstrating free air in the mediastinal space indicative of pneumomediastinum CT: computed tomography

Serial troponins and D-dimer were found to be negative. Given severe pleuritic chest pain, CT pulmonary angiogram (CTPA) and CT aortogram were performed, which showed the presence of free air in the mediastinum without an obvious cause (Figure 2); however, no pneumothorax, pulmonary embolism, or acute aortic pathology was identified. A gastrografin swallow test was performed as an additional diagnostic test to find any esophageal perforation, and it returned negative. In keeping with a diagnosis of SPM, no further investigation was planned. The patient was managed conservatively with simple analgesia and kept on telemetry for monitoring of vital signs. He was consistently observed to have heart rates in the lower 40s without any signs of hemodynamic compromise. Over the period of hospital stay, he made a good recovery. Chest pain and breathlessness improved alongside gradual spontaneous resolution of bradycardia. His heart rate was recorded stable at around 65 on subsequent follow-up. He was advised against scuba diving and Valsalva maneuvers and discharged home with a respiratory clinic follow-up appointment in three months.

## Discussion

While pontaneous pneumomediastinum is an extremely rare condition in the general population, a relatively higher prevalence is observed among the young adult age group. Chest pain and dyspnea are the most common presenting symptoms described; nevertheless, a few studies have reported bradycardia as a manifestation in rare cases, which could have been due to excessive vagal stimulation related to increased stimulation of receptors in the aortic arch and/or carotid sinus by the trapped gas in the mediastinum. Irwin et al. first reported a case of SPM with asymptomatic bradycardia along with left ventricular hypertrophy (LVH) and Wellens' signs in ECG [2]. Nischal et al. have also described patients presenting with SPM associated with asymptomatic bradycardia [3].

Our patient had considerable sinus bradycardia on admission. It was presumed to be SPM-related given his unremarkable comprehensive bradycardia workup, and it eventually resolved together with the improvement in other presenting symptoms. In terms of imaging, chest radiographs are likely to be diagnostic in most cases of pneumomediastinum; however, chest radiography of a significant proportion of patients could be found negative and they may require CT to confirm a diagnosis. Caceres et al. published a comparative study where a chest radiograph was diagnostic in 69% of cases, while additional CT was required to reach the diagnosis in the remaining 31% [4]. In a retrospective review of radiographic pneumomediastinum cases by Bakhos et al., CT was performed in 38 patients and showed a pneumomediastinum in nine patients, which was not seen on chest X-rays [5].

Additionally, in a retrospective review spanning years, Park et al. reported that chest X-ray findings were diagnostic in 48.4% of confirmed cases of SPM [6]. Our patient’s chest X-ray was unremarkable on admission; however, subsequent CT showed pneumomediastinum. Furthermore, asymptomatic sinus bradycardia at presentation was even more unusual, a phenomenon that has been observed only in very few cases of SPM in the published literature.

## Conclusions

SPM is a rare entity and is usually benign. However, it should be included in the differential diagnosis of acute chest pain, particularly in the young adult age group of patients, who have a relatively higher incidence of the condition. Of note, SPM can develop without a triggering event and may even have normal chest radiography on assessment, as in our case. Therefore, to avoid a missed diagnosis, a CT scan of the chest should be considered earlier in patients with a high clinical suspicion of pneumomediastinum despite negative findings on chest X-ray. Additionally, bradycardia could be a rare manifestation of SPM; it is usually asymptomatic and resolves with the improvement of the primary condition. Nevertheless, patients should be monitored for haemodynamic stability and relevant investigations should be performed to exclude other causes of bradycardia.
